# *Brevibacterium* Bacteremia in the Setting of Pyogenic Liver Abscess: A Case Report with Accompanying Literature Review

**DOI:** 10.1155/2021/8034874

**Published:** 2021-12-28

**Authors:** Sarah Hossain, Afif Hossain, Aldo Barajas-Ochoa, Michael A. Jaker

**Affiliations:** ^1^American University of Antigua, Jabberwock Beach Road, PO Box W1451, Coolidge, Antigua and Barbuda; ^2^Department of Internal Medicine, Rutgers New Jersey Medical School, 185 S Orange Avenue, Newark, NJ 07103, USA

## Abstract

A 71-year-old Pakistani man with poorly controlled type 2 diabetes mellitus presenting with worsening mental status, abdominal pain, and oral intake for the past seven days was found to have pyogenic hepatic abscess with unculturable bacteria and subsequently found to have rare *Brevibacterium* bacteremia.

## 1. Introduction

*Brevibacterium* is a short coryneform species found in dairy products and known colonizers of the human skin. Early in its life cycle, *Brevibacterium* exhibits typical features of coryneform bacteria. However, as it matures, it takes on an appearance similar to cocci or coccobacilli [1]. The most common species isolated from humans is *Brevibacterium casei* [2], which appears as a catalase-positive, non-spore-forming, short, club-shaped rod on gram staining. Most commonly, bacteremia is associated with indwelling intravascular catheters in the immunocompromised [3]. However, there are rare cases leading to meningitis, cholangitis, salpingitis, peritonitis, endocarditis, and osteomyelitis. The treatment of choice for serious infections is vancomycin as the bacteria show some resistance to B-lactams, fluoroquinolones, clindamycin, and macrolide antibiotics [4]. To date, there are only 16 published case reports of *Brevibacterium* bacteremia [5].

## 2. Case Presentation

A 71-year-old Pakistani man presented to University Hospital in Newark, NJ, in May of 2020 after being brought in by emergency medical services from home for worsening mental status over the last seven days. The patient had a history of triple vessel coronary artery disease after percutaneous coronary intervention and stenting to the acute marginal branch of the right coronary artery in June, 2018, balloon angioplasty to the proximal and middle right coronary artery in 2011, and stenting of the left anterior descending artery in 2011, poorly controlled type 2 diabetes mellitus, hypertension, and hyperlipidemia. According to the patient's family, he had worsening mental status, decreased oral intake, and worsening abdominal pain over the last week, with a sharp decline over the past two days.

At baseline, the patient was able to perform all activities of daily living independently, was able to hold full conversations in English and Urdu, and had no issues with ambulation. He had no other symptoms prior to arrival. Furthermore, the patient was not taking his medications regularly, including insulin. For diabetes, the patient was previously prescribed insulin, metformin, glimepiride, canagliflozin, and sitagliptin. However, it was unclear which of these medications he was indeed taking as he had not been seen outpatient in over 10 years. Of note, the patient had traveled to Pakistan and Dubai within the last year but did not have any sick contacts.

**Figure 1:**
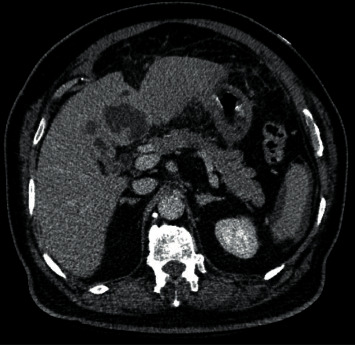
Computed tomography of the abdomen and pelvis with intravenous contrast in the transverse section showing rim-enhancing fluid-filled collections in hepatic segments 4A and 4B, 6 cm by 4.6 cm in size in the greatest axial dimensions. There are several smaller, localized satellite lesions. Findings are highly suspicious for a liver abscess.

In the emergency room, the patient was found to be febrile to 101.8⁰F, tachycardic to 128, normotensive, tachypneic to 20, and saturating 99% on room air. On exam, he was disoriented and confused, speaking incoherently in one-word sentences, had poor dentition with dry mucous membranes, tachycardic, and had diffuse abdominal tenderness to minimal palpation, worse in the right upper quadrant but without rebound or guarding. Admission labs were significant for a leukocytosis of 12, hemoglobin of 8.3 from a baseline of 12 with a mean corpuscular volume of 63.1, sodium of 124, bicarbonate of 18, glucose of 399 but negative acetone, anion gap of 18 with lactic acidosis to 3.5, HbA1c 12.2, procalcitonin 8.16, c-reactive protein 50, negative cardiac enzymes, and normal liver enzymes. Given his altered mental status and severe sepsis, the patient had computed tomography of the abdomen and pelvis with intravenous contrast carried out (Figure 1), showing multiple colonic diverticula without diverticulitis and a 6 by 4.6 centimeter rim-enhancing lesion with surrounding satellite lesions, likely representing a liver abscess.

The patient was started on aggressive intravenous fluid resuscitation, morphine for pain control, and intravenous Flagyl (metronidazole) and cefepime for 2 days. Vancomycin was later added to cover for *Enterococcus* but discontinued after three doses, so no level was checked. COVID-19 testing, interferon-*γ* release assay, *Entamoeba histolytica*, and *Echinococcus* serologies were all negative. During his hospital course, the patient had uncontrollable blood sugars and ultimately required 65 units of Lantus daily, 8 units of lispro with meals, and an additional 20–25 units of coverage daily. Abscess drainage by interventional radiology was performed on admission and then again 6 days later. Two sets of body fluid cultures were sent, which grew Gram-positive cocci in pairs and chains but could not be speciated. Blood cultures eventually speciated *Brevibacterium* from aerobic bottles twice. Unfortunately, real-time polymerase chain reaction was unable to identify speciation for the causative organism for either set of body fluid cultures or blood cultures. Infectious Disease was consulted, and the patient was switched to ampicillin-sulbactam (Unasyn) 3 g every six hours for a minimum 3-week course with a plan for repeat imaging after 3 weeks of intravenous antibiotics. After a 10-day hospital stay and improvement in his condition, the patient was safely transferred to subacute rehabilitation for continued antibiotic therapy.

## 3. Discussion and Literature Review

To date, there are only 18 publications mentioning *Brevibacterium* bacteremia, with 16 of those cases published as case reports [5]. The earliest publication, dating back to 1973, involved pediatric patients undergoing carious extraction for moderate to severe dental pathology [6]. Blood cultures were drawn several times pre- and postoperatively, with one of thirty-four patients testing positive for Brevibacterium. In Saudi Arabia, a 2004 study found *Brevibacterium* bacteremia in four critically ill patients, highlighting its involvement in serious infections in the immunocompromised [7]. From the published case reports, 11 speciated *Brevibacterium casei*, 1 *Brevibacterium epidermidis*, 1 *Brevibacterium paucivorans*, 1 *Brevibacterium massiliense*, and 2 were not specified. Seven patients had underlying malignancy, two had AIDS, five had chronic medical comorbidities, one had a congenital abnormality of metabolism, and one was not mentioned. 13 patients had some form of indwelling catheter, 2 were not specified, and 1 had no indwelling catheter present. The majority of patients were given broad-spectrum antibiotics, including vancomycin, teicoplanin, aminoglycosides, extended-spectrum beta lactams, and/or fluoroquinolones. 14 patients improved, 6 patients had recurrence, and 2 patients died. See Table 1 for further details.

As mentioned previously, *Brevibacterium* are nonmotile, non-spore-forming, non-acid-fast, obligate aerobes. They are Gram-positive bacilli in singlets, pairs, or short chains which transform into cocci approximately 48 hours after inoculation. Characteristic features include positivity for lipase, catalase, DNAse, litmus milk, oxidase variability, urease negativity, and reduction of nitrates to nitrites [1]. Though there are various strains, *Brevibacterium casei* is the most common clinically relevant pathogen [2]. *Brevibacterium* species are typically innocuous bacteria found in cheeses, poultry, and human skin flora. However, in the immunocompromised and, especially, those with indwelling catheterization [3], they can function as devastating opportunistic pathogens. In addition to the aforementioned bacteremia cases, they rarely can cause brain abscess, meningitis, endocarditis, pericarditis, peritonitis, cholangitis, salpingitis, mastitis, and osteomyelitis. Although vancomycin is used most frequently, ultimately the antibiotic chosen depends on the infection site and severity of infection [4].

In this case report, a patient with pyogenic liver abscess was found to have concomitant *Brevibacterium* bacteremia. To our knowledge, this is the first instance of such a phenomenon and one of the only cases without central-line-associated infection. It is unclear if our patient had bacterial translocation from colonic diverticula leading to hepatic abscess. Another potential source is yogurt and cheese consumed by the patient while abroad in Pakistan and/or Dubai, which is a common delicacy with most meals. Despite very poorly controlled diabetes, the patient showed improvement in his condition after abscess drainage and treatment with intravenous Unasyn.

Despite prior notions that *Brevibacterium* species pose little to no harm clinically, evolving evidence points towards the contrary. Given the severity of bacteremia cases in the immunosuppressed, *Brevibacterium* can function as a serious and deadly causative opportunistic agent. Utilization and maintenance of long-term indwelling catheters requires close adherence to sterile technique. Earlier case reports highlight non-specific symptomatology, with an often indolent presentation, which later manifests as florid septicemia. In these cases, prompt initiation of broad-spectrum, empiric antibiotics can be lifesaving but should later be deescalated based on the source of infection and susceptibilities in accordance with proper antibiotic stewardship.

## 4. Conclusions

*Brevibacterium* infection is an uncommon but potentially fatal cause of bacteremia in the immunocompromised. This risk is increased with prolonged use of indwelling catheters and implanted devices. Infections are often indolent initially but can rapidly escalate if left untreated. Though initial treatment typically includes broad-spectrum antibiotics, treatment can be de-escalated based on the source of infection and susceptibilities with favorable outcomes. Our report hopes to provide further insight into the management of *Brevibacterium* bacteremia, specifically in the setting of pyogenic liver abscesses, for which there are no documented cases.

## Figures and Tables

**Table 1:** Clinical summaries of *Brevibacterium* bacteremia case reports.

Author (year)	Sex	Age	*Brevibacterium* species	Underlying condition	Clinical course	Treatment regimen (duration)	Presence of an indwelling catheter	Outcome
McCaughey and Damani (1991) [8]	M	40	*epidermidis*	Zollinger–Ellison Syndrome	Vomiting, weight loss, recurrent duodenal ulceration, pyloric outflow obstruction	Erythromycin, TLC^a^ removal	Yes- indwelling subclavian TLC^a^ for TPN^b^	Survived
Lina et al. (1994) [9]	M	19	Not specified	Lymphoblastic Lymphoma	Fever, retroocular pain; recurrence after 1 month	IV teicoplanin, amikacin for 20 days; teicoplanin x21 days, TLC^a^ removal	Yes- for chemotherapy; type not specified	Recurrence; survived
Reinert et al. (1995) [10]	M	25	*casei*	Testicular choriocarcinoma	Fever, pancytopenia	IV piperacillin, teicoplanin for 10 days; piperacillin, tobramycin for 10 days	Yes- TLC^a^ for chemotherapy	Recurrence; survived
Kaukoranta-Tolvanen et al. (1995) [11]	F	56	*casei*	Non-Hodgkin lymphoma	Fever, pancytopenia, CRP^c^ 42 mg/dL	Not specified	Yes- TLC^a^ for chemotherapy	Recurrence; survived
Castagnola et al. (1997) [12]	---	---	*casei*	Neuroblastoma	Fever, ANC^d^>1000 cm^3^	Not specified; TLC^a^ removed	Yes- Broviac® for chemotherapy	Survived
Brazzola et al. (2000) [13]	F	18	*casei*	Acquired Immunodeficiency Syndrome (AIDS)	Fever, dehydration	IV Unasyn, switched to ciprofloxacin for 14 days; TLC^a^ removed	Yes- Port-A Cath® for PPN^e^	Survived
Ogunc et al. (2002) [14]	---	60	Not specified	Chronic Lymphocytic Leukemia (CLL)	Fever following fludarabine chemotherapy, anemia	IV ceftazidime, amikacin; switched to vancomycin	Not specified	Survived
Janda et al. (2003) [7]	M	34	*casei*	Acquired Immunodeficiency Syndrome (AIDS)	CD4<50, known CMV retinitis, oropharyngeal candidiasis, neutropenic fever, malaise	IV vancomycin for 8 days, ceftazidime (stopped); TLC^a^ removed	Yes- Hickman® catheter for long-term gancylovir infusion	Survived
Beukinga et al. (2004) [15]	F	43	*casei*	Crohn's Disease	Chronic fistulae, total colectomy, fever, WBC^f^ 3300, CRP^c^ 5.8 mg/dL	IV vancomycin for 15 days, TLC^a^ remained; IV Unasyn, Merrem, amikacin, TLC^a^ removed	Yes- Port-A Cath® for PPN^e^	Recurrence (at 5 months); died
Beukinga et al. (2004) [15]	M	31	*casei*	Not specified	Fever, WBC^f^ 4700, CRP^c^ (-)	IV vancomycin for 15 days, TLC^a^ remained; same treatment	Yes- Hickman® catheter for hemodialysis	Recurrence (at 5 months); survived
Ulrich et al. (2006) [3]	F	62	*casei*	Severe pulmonary hypertension	Flu-like symptoms, productive cough, chills, fever, hypoxemia, CRP^c^ 38 mg/dL	IV vancomycin for 10 days, then moxifloxacin for 20 days, TLC^a^ removed	Yes- TLC^a^ for iloprost infusion	Survived
Bal et al. (2015) [16]	M	6	*casei*	Acute Lymphoblastic Leukemia (ALL), B cell type	Herpes zoster infection, pancytopenia, neutropenic fever, ANC^d^ 387 mm3/uL, CRP^c^ 6.1 mg/dL	IV Zosyn, vancomycin for 10 days, TLC^a^ remained	Yes- Hickman® catheter for chemotherapy	Survived
Bonavila Juan et al. (2017) [17]	M	60	*casei*	Child–Pugh C alcoholic cirrhosis, aortic stenosis; development of aortic valve endocarditis and insufficiency with recurrence	Tremor, altered mental status, fever, pustular rash; decompensated cirrhosis, coagulopathy, thrombocytopenia, 1.5 cm aortic valve vegetation seen on TEE^g^; right arm weakness, septic emboli	Oral Levaquin for 10 days, then norfloxacin; IV vancomycin for 4 weeks; IV vancomycin for 10 days, daptomycin for 6 days	Not specified	Recurrence (30 days, 90 days); died
Vecten et al. (2017) [18]	M	4	*massiliense*	Congenital methylmelonic acidemia	Fever, cough, emesis, left ear discharge, WBC^f^ 9400uL, CRP^c^ (-), oxalic acid 0.020 mmol/L	Intra-auricular ofloxacin for 8 days	Yes- gastrostomy tube present	Survived
Magi et al. (2018) [19]	F	48	*Casei*	Bilateral breast cancer requiring mastectomy, chemotherapy, radiation, and salpingoophorectomy	Fever, myalgia, CRP^c^ 5.97 mg/dL	IV teicoplanin for 7 days, linezolid for 7 days, TLC^a^ removed	Yes- transjugular Port-A-Cath® from prior chemotherapy treatment	Survived
Asai et al. (2019) [5]	F	94	*paucivorans*	Type 2 diabetes mellitus, congestive heart failure	Fever, decreased oral intake, appetite loss, thrombocytopenia, CRP^c^ (-)	IV Merrem, teicoplanin for 14 days	Not present	Survived
Our Case	M	71	Unable to speciate	Poorly controlled type 2 diabetes mellitus, pyogenic liver abscess	Altered mental status, abdominal pain, decreased oral intake, WBC^f^ 12,000, anemia, hyponatremia, hyperglycemia, HbA1c 12.2, acetone (-), CRP^c^ 50 mg/dL, procalcitonin 8.16	IV Unasyn for 3 weeks, abscess drainage	Not present	Survived

M = male, F = female; ^a^TLC = triple lumen catheter; ^b^TPN = total parenteral nutrition, ^c^CRP = c-reactive protein, ^d^ANC = absolute neutrophil count, ^e^PPN = partial parenteral nutrition, ^f^WBC = white blood cell, ^g^TEE = transesophageal echocardiogram.

## Data Availability

No data were used to support this study.

## Abbreviations

COVID-19: Coronavirus disease 2019
M: Male
F: Female
TLC: Triple lumen catheter
TPN: Total parenteral nutrition
CRP: c-reactive protein
ANC: Absolute neutrophil count
PPN: Partial parenteral nutrition
WBC: White blood cell
TEE: Transesophageal echocardiogram.
